# The psychiatry resident research experience

**DOI:** 10.1186/s13104-016-2290-1

**Published:** 2016-11-14

**Authors:** Frank P. MacMaster, Jordan Cohen, Waqar Waheed, Emilie Magaud, Mariko Sembo, Lisa Marie Langevin, Katherine Rittenbach

**Affiliations:** 1 Psychiatry, University of Calgary, Calgary, Alberta Canada; 2 Pediatrics, University of Calgary, Calgary, Alberta Canada; 3 Hotchkiss Brain Institute, Mathison Centre for Mental Health Research & Education, Calgary, Alberta Canada; 4 Child and Adolescent Imaging Research Program, Alberta Children’s Hospital Research Institute, Calgary, Alberta Canada; 5 Strategic Clinical Network for Addictions and Mental Health, Alberta Health Services, Edmonton, Alberta Canada; 6 Child and Adolescent Imaging Research Program, University of Calgary, Alberta Children’s Hospital Research Institute, 2888 Shaganappi Trail NW, Calgary, AB T3B 6A8 Canada

**Keywords:** CanMEDS, Psychiatry, Research, Residents, Scholar

## Abstract

**Background:**

Research activity is especially critical in the field of psychiatry as it is evolving rapidly thanks to advances in neuroscience.

**Results:**

We administered a 34-item survey regarding research experiences targeted at psychiatry residents and postgraduate residency program directors in Canada. One hundred and nineteen participants answered the survey (16 program directors, 103 residents) allowing for a margin of error of 8.4% at a 95% confidence interval. Research was rated as important in informing clinical practice (87.0% yes, 13.0% no), but only 28.7% of respondents reported that it was taught well at their home institution (33.0% no, 38.3% neutral). Only a small proportion was enthusiastic or very enthusiastic about participating in research (21.7%).

**Conclusions:**

While the importance of research is recognized, there is little consensus with respect to whether a standardized research practicum component is included in the resident curriculum.

**Electronic supplementary material:**

The online version of this article (doi:10.1186/s13104-016-2290-1) contains supplementary material, which is available to authorized users.

## Background

Residency training prepares physicians to practice medicine independently. Research and scholarly activity is included in this training as it benefits the residents, their patients, and the health care system [1]. In Canada, the Royal College of Physicians and Surgeons CanMEDS Physician Competency Framework states, “As Scholars, physicians demonstrate a lifelong commitment to reflective learning, as well as the creation, dissemination, application and translation of medical knowledge”. In practice, how residency programs incorporate research into their training requirements varies greatly, and is especially critical in the field of psychiatry as it rapidly evolves thanks to advances in neuroscience [2]. The goal of the survey was to gain an understanding of what the standard of practice is for research training across Canada, by assessing the perceptions of residents and their program directors within those programs. Specifically: (1) to describe how psychiatry residency programs fulfill research activity requirements, (2) to describe resident and residency director attitudes towards research, and (3) to describe barriers and enablers towards participating in research components during residency.

Our basic conceptual framework [3] is first to understand the resident’s perception of research within their personal experience. To do this, we developed a survey based on previously published studies in similar samples [4–8] that would allow for the identification of broad trends in residents’ perceptions of research regarding their knowledge, attitudes, and practices.

## Results

In keeping with the approach outlined by Glassick [9], the survey was adapted from previous studies [4–8] and uploaded on Survey Monkey in English and French. Requests to participate were sent to program directors (N = 17 for psychiatry and N = 12 for subspecialty in child and adolescent) and psychiatry residents (N ~ 893) across Canada. Participants logged onto the survey site and consent was considered as having been granted if they chose to participate in the survey after presentation of the study information sheet. The survey typically took less than 20–25 min to complete. As an incentive for completing the survey, subjects could provide their email for a draw of a gift card ($25). The Conjoint Health Research Ethics Board (CHREB) of the University of Calgary provided ethical approval for this study. An Excel file of the raw data can be made available by emailing the corresponding author. When possible, the responses of program directors and residents were separated.

One hundred nineteen participants completed the survey: 16 program directors (55.0%) and 103 residents (13.0%). With an overall population of N = 922, this established a margin of error of 8.4% at a 95% confidence interval. Of the residents who responded, 24.3% were in first year, followed by 20.4, 14.6, 23.3, and 17.5% in years 2–5 respectively. Almost half of respondents indicated they were not pursuing a subspecialty (46.2%). Of reported subspecialties, 23.1% indicated child and adolescent, 4.3% geriatric, 6.8% forensic, with 19.7% were undecided at the time of the survey.

An overwhelming majority of respondents considered research important for informing their critical practice (see Table 1 for a summary of selected results; broken down—93.8% of program directors and 82.5% of residents agreed or strongly agreed). However, only 56.3% of program directors and 23.3% of residents agreed or strongly agreed that research was taught well at their home institute. This contrasts with the fact that over half of respondents overall felt the emphasis on research was high in their department (75.1% of program directors and 48.5% of residents). Participation in research was considered mandatory by 81.3% of program directors and only 44.7% of residents. A small proportion of residents were enthusiastic (17.5%) or very enthusiastic (1.0%) about research, with 59.2% somewhat enthusiastic and 19.4% not enthusiastic at all.

**Table 1:** Summary of survey results

Question	Yes (%)	No (%)	Don’t know (%)
Is research important in informing your clinical practice?	87.0	13.0	–
Is research taught well?	28.7	71.3	–
The emphasis on resident research in your department is high?	54.9	45.1	–
Is participation in research mandatory?	49.6	45.4	5.0
Does you program have a research director?	91.5	2.5	5.9
Do you receive adequate training in reviewing medical literature?	63.9	36.1	–
Do you receive adequate training in research design?	40.3	59.7	–
Do you receive adequate training in methodology?	34.5	65.5	–
Do you receive adequate training in grant applications?	7.6	92.4	–
Do you receive adequate training in writing papers?	7.6	92.4	–
Do you receive adequate training in publishing?	6.7	93.3	–
Do you receive adequate training in presentations?	32.8	67.2	–
Is there was a good match between available resident time and researcher expectations?	62.3	37.7	–
Is research time protected?	70.6	19.3	10.1
Do funding opportunities exist to support resident research?	76.5	7.5	16.0

With regard to program structure, 89.3% of residents and 100% of program directors indicated their program had a research director. Regarding to whom the resident was “most accountable to regarding research”, 29.1% of residents said their research mentor, 21.4% said their program’s research coordinator/director, 14.6% said the program director, 25.2% said they did not know, and 9.7% stated no one in particular. Interestingly, 43.8% of program directors stated their program’s research coordinator/director, 37.5% the research mentor, 18.8% themselves.

Questions regarding methodology of research teaching showed that research design, methods, and statistics were most commonly taught in a formal lecture series (78.2%). Other forms of teaching included: “as needed by faculty in the program” (56.3%), “as needed by faculty outside the program” (15.1%), or in journal clubs (40.3%). Program directors differed from residents in endorsement only with regard to journal clubs (62.5 and 36.9% respectively). A small minority of respondents (1.7%) said such teaching was not available, and another small group (5%) did not know how or if these subjects were taught.

A majority of respondents stated residents received adequate training in reviewing medical literature (87.5% of program directors and 60.2% of residents), enabling them to assess the validity of new discoveries and the applicability of findings to their practice. However, less than a majority endorsed sufficient training in activities required to complete their own research. Most residents felt there was a good match between available resident time and researcher expectations (62.3; 75.1% of program directors and 57.3% of residents). Further to this, most respondents (70.6; 87.5% of program directors and 68.0% of residents) said research time was protected.

Most participants rated their residency program as being about the same (47.8; 50.0% of program directors and 44.7% of residents) or better (31.8; 31.3% of program directors and 30.1% of residents) compared to other programs when it came to fostering resident research. They considered the research productivity of residents in their program as being moderate (46.5; 50.0% of program directors and 43.7% of residents), low or very low (28.9; 18.8% of program directors and 48.0% of residents), and high or very high (24.6; 25.0% of program directors and 23.3% of residents). Interestingly, they viewed their department’s productivity more positively, as it was ranked as high or very high (44.7; 31.3% of program directors and 45.7% of residents), slightly more than moderate (40.4%), and only 14.9% as low or very low.

A majority also considered the faculty as being qualified or very qualified to teach principles of research (70.2; 75.0% of program directors and 66.1% of residents) while 28.9% viewed their faculty as only “somewhat qualified” (18.8% of program directors and 29.1% of residents, with 0.9% saying their faculty was “not at all qualified” (all residents). Most considered faculty accessible (62.6% of program directors and 71.9% of residents) and supportive (87.6% of program directors and 74.8% of residents) to residents interested in research, with sufficient faculty mentors available (75.0% of program directors and 60.2% of residents). A majority (62.6% of program directors and 64.1% of residents) felt the faculty had sufficient time to help residents with research, with enough technical support (i.e., ethics, biostatistics, writing) (62.6% of program directors and 57.3% of residents).

Mentoring was received from a designated research advisor (67.2; 87.5% of program directors and 64.1% of residents), individual faculty in the program (68.9; 68.8% of program directors and 70.9% of residents), or individual faculty outside the program (22.7; 31.3% of program directors and 21.4% of residents); 5% reported they did not know (all residents). Many stated there was no formal mentor program (i.e., it just ‘happens’ based on mutual interest, 42.4; 18.8% of program directors and 45.6% of residents) or a combination of formal and informal mentoring (31.4; 43.8% of program directors and 29.1% of residents), with formal mentoring accounting for 16.1% (31.3% of program directors and 13.6% of residents); 10.1% said they did not know (all residents).

In agreement with the findings that writing papers and publishing were not commonly taught, most residents did not know (26.2%) or thought there was no requirement (33.0%) for outputs of their research. A quarter of respondents were expected to present their project/research at rounds (25.2; 37.5% of program directors and 23.3% of residents) or other meeting (26.9; 50.0% of program directors and 23.3% of residents). However, academic/research writing was less frequently expected: case reports—11.8% (25.0% of program directors and 9.7% of residents), review articles—17.6% (37.5% of program directors and 14.6% of residents), and original research articles 16.0% (37.5% of program directors and 12.6% of residents). Most residents did not know if there were any ramifications if they failed to meet this requirement (57.3%), while almost a third (31.3%) of program directors said no policy exists at their institutions. In contrast, 31.3% of program directors and 8.7% of residents also said residents were not permitted to graduate unless this research requirement was attained. For 17.6% it was not applicable, as they did not have an output requirement. This means a substantial number of those without a requirement still generated research output. Among respondents, possible reasons for not having a requirement included the feeling that research should be optional (24.4; 12.5% of program directors and 26.2% of residents), too busy (10.9; 12.5% of program directors and 10.7% of residents), residents would object (5.9; 6.3% of program directors and 5.8% of residents), and insufficient faculty mentors (4.2%; 6.3% of program directors and 3.9% of residents).

Over three quarters (76.7%) of residents and program directors (75%) reported that funding opportunities exist for research. Overall, when broken down, 43.7% said funding for research came from grants belonging to faculty members (75.0% of program directors and 38.8% of residents), and 37.0% said departmental funds (68.8% of program directors and 32.0% of residents). More than a quarter (27.7%) said they had to apply for grants themselves, either with the help of the program or without (21.0; 43.8% of program directors and 25.2% of residents).

If a resident succeeds in developing a hypothesis and carrying it to publication, an overwhelming majority felt they received proper credit (87.5; 100% of program directors, 80.6% of residents). When asked if guidelines existed in their programs to determine authorship, and who would be funded to present at meetings, most did not know (80.7%); only a fraction endorsed that their programs had any explicit guidelines.

## Discussion

On balance, this survey demonstrates that residency training in psychiatry is currently creating a greater number of passive consumers of research. Indeed, the next generation of clinician scientists is not being well prepared to generate new knowledge and apply it proactively within their respective practices.

While participants considered research as important for informing clinical practice, this did not translate to enthusiasm to participate in research as residents. More than three quarters of respondents stated residents were only somewhat enthusiastic or not enthusiastic at all about research. This lack of enthusiasm is a critical barrier in improving research training. It is not surprising given that the emphasis on research was equivocal within departments of psychiatry. The nature of the enthusiasm deficit for research requires further investigation. Almost half said participation in research was considered mandatory, which is in keeping with previous work indicating that 32% of respondents indicating that it should be a requirement [10].

The infrastructure needed for resident participation in research is strong. Most programs have a research director, abundant faculty mentors, adequate and protected time, and financial support for research efforts. However, lack of formal mentoring programs, clear accountability, along with clear and achievable expected outputs are barriers to resident participation in research. In addition, the lack of training in grant writing may prevent residents from accessing available support.

The concern over the quality of research teaching was possibly the most critical concern. Indeed, aside from reviewing literature, training in common aspects of research (papers, methodology, research design, publishing, presentations) is profoundly lacking. This may be easily remedied however by providing templates of papers, grants, and presentations (poster, oral) and developing specific support mechanisms (i.e., internal review) for grant applications (see Table 2). There is also a lack of clarity and consistency among programs regarding expected deliverables when it comes to research proficiency. The demands on time from training may create a negative bias towards becoming involved in research and executing projects that are more substantial.

**Table 2:** Recommendations for incorporating research into residency training in psychiatry

Level	Output	Elements	Support	Adjudication
*Knowledgeable consumer of research*	Systematic review	Question/PICO	Guidelines and templates on PICO development	Submission for review within the faculty or publication (peer review)
		Literature Search	Guidelines on process	
		Critical Evaluation	Guidelines on evidence	
		Synthesis	Mentorship on interpretation	
		Manuscript	Templates	
	Presentation	Oral or poster	Templates and opportunity for rehearsal/practice	Acceptance at rounds or scientific conference
*Creator of new knowledge*	Original research	Question/PICO	Guidelines and templates on PICO development	Submission for review within the faculty or publication (peer review)
		Literature search	Guidelines on process	
		Critical evaluation	Guidelines on evidence	
		Study design	Mentorship, guidelines, and templates; timeline development	
		Ethics	Mentorship, guidelines, and templates	
		Funding	Mentorship, guidelines, and templates	
		Data collection	Mentorship and technical support	
		Data analysis	Mentorship and technical support	
		Data interpretation	Mentorship and technical support	
		Manuscript	Templates	
	Presentation	Oral or poster	Templates and opportunity for rehearsal/practice	Acceptance at rounds or scientific conference

CanMEDS incorporates research into two milestones: first, as a consumer, it expects residents to be able to “critically evaluate the integrity, reliability, and applicability of health-related research and literature”. Second, as a creator of knowledge, it expects residents to be able to “contribute to the dissemination and/or creation of knowledge and practices applicable to health”. The milestone for the Accreditation Council for Graduate Medical Education (ACGME) has a broader stance but is essentially similar in nature, expecting the “development and execution of lifelong learning through constant self-evaluation, including critical evaluation of research and clinical evidence”. More practically, these two roles (consumer and creator) can be broken down to key subcomponents (see Table 2), that need to be delivered in a deliberate planned program, as opposed to the more common laissez-faire approach currently in place across Canada. In response to our data presented here, we propose a “Three E” process to address this: the “Three E’s” are to develop (1) enthusiasm for research, (2) education on research process, and (3) support in the execution of a research project (Additional file 1).

As stated earlier, to address the lack of enthusiasm for research among residents, further research is needed (i.e., focus groups). With adequate knowledge as to the underlying rationale, the barriers can be addressed. Regarding education on research process, information must be made accessible to residents in a manner they can easily access and consume. For example, developing templates for research papers, posters, abstracts, ethics applications, etc. and making them available online so that they can be accessed as needed by residents. These templates would include guide text and examples, to help shape the research output. For the execution of a research project—be it a systematic review or an original research study—mentorship, clear expectations, and benchmarks are needed to promote success. All too often, poor planning of timelines can scuttle a project and taint the research experience for a resident.

Limitations of this study include the fact that as a survey, responses are based off the participant’s perception of their training environment, and even the intent of the questions themselves. In addition, the low response rate for residents and program directors can introduce bias. However, our margin of error of 8.4% at a 95% confidence interval is in keeping with similar surveys. The nature of the responses was in keeping with previous studies, which leads us to believe our study is representative [10]. Comparing program directors directly to residents is of great interest and has been done previously [10]. Our study design did not lend itself to a robust level of analysis however and should be viewed as more speculative in that regard. A further limitation is the distribution across general psychiatry and subspecialties. We feel that this study provides a vital window into how residents and program directors perceive research and its current status in their training.

## Conclusion

There is growing concern that clinician scientists are disappearing [11], and they are sorely needed in psychiatry. Transformative innovation and paradigm shifts are occurring in psychiatry, and the tools are found in neuroscience, epidemiology, and health economics. To allow participation in this shift, we must invest resources in people to encourage their participation. There are numerous alternative career paths for potential clinician scientists that lure top minds away. Furthermore, the PhD scientist process is one of concentrated apprenticeship with little competing demands, while residents face the daunting tasks of mastering their clinical skills while being expected to engage in research. The effective practice of psychiatry will be increasingly dependent on research. As such, clinicians need to be familiar with research methods to both interpret and apply the developing psychiatric literature. If progress in psychiatry is to be made, we need to invest in a culture of innovation in addition to care delivery. The ever-growing pressure on delivery of care has constrained research activity and created obstacles in the translation of research findings to the clinic. We need a robust and active generation of clinician-scientists to lead the way.

## Additional file

**Additional file 1.** Copy of the questionnaire used in the study.

## Abbreviations

ACGME: Accreditation Council for Graduate Medical Education
CHREB: Conjoint Health Research Ethics Board
