# Supplementary material for: The psychiatry resident research experience
Source: BMC Res Notes. 2016 Nov 14;9:486. doi: 10.1186/s13104-016-2290-1 (PMC5109781; doi:10.1186/s13104-016-2290-1)
Supplement: Supplementary file 1 — Additional file 1. Copy of the questionnaire used in the study. [file 13104_2016_2290_MOESM1_ESM.pdf]

Demographics

1. What is your position?

- ◯ Program Director
- ◯ Resident
- ◯ Child and Adolescent Subspecialty

Other (please specify)

2. Residency year?

- ◯ Not applicable
- ◯ 1
- ◯ 2
- ◯ 3
- ◯ 4
- ◯ 5

3. Institution?

- University of British Columbia
- University of Calgary
- University of Alberta
- University of Saskatchewan
- University of Manitoba
- Northern Ontario School of Medicine
- Western University
- McMaster University
- University of Toronto
- Queen's University
- University of Ottawa
- McGill University
- Université de Montréal
- Université de Sherbrooke
- Université Laval
- Dalhousie University
- Memorial University of Newfoundland
- Other (please specify)

4. Do funding opportunities exist to support resident research?

- Yes
- No
- Don't know

5. Residents are taught about basic research design, methods, and statistics: (Select all that apply)

- [ ] In formal lecture series
- [ ] As needed by individual faculty within the program
- [ ] As needed by faculty outside the program
- [ ] As a formal part of journal clubs
- [ ] No such support available
- [ ] Don't know

6. Does your program have a research director or coordinator?

- ○ Yes
- ○ No
- ○ Don't know

7. Is participation in research mandatory?

- ○ Yes
- ○ No
- ○ Don't know

8. Residents in your program get research mentoring advice from: (Select all that apply)

- [ ] A designated research advisor
- [ ] Individual faculty members
- [ ] Faculty from other departments
- [ ] No mentoring available
- [ ] Don't know

9. Is it a formal or informal research mentoring program?

- ○ Yes - mentor and mentee are assigned to each other with the help of the director/coordinator
- ○ No - it just 'happens" based on mutual interest
- ○ Both
- ○ Don't know

10. To whom is the resident most accountable in regards to research projects?

- ○ Research Mentor
- ○ Director or Coordinator of Research
- ○ Residency Program Director
- ○ Department Chair
- ○ No one in particular
- ○ Don't know

11. Is research time protected?

- ○ Yes
- ○ No
- ○ Don't know

12. Do residents in your program get adequate training in: (Select all that apply)

- ☐ Reviewing the literature
- ☐ Research design
- ☐ Methodology
- ☐ Grant applications
- ☐ Writing papers
- ☐ Getting published
- ☐ Presentations

13. What is your current or planned subspecialty, if any?

- ○ None
- ○ Child and Adolescent
- ○ Geriatric
- ○ Forensic
- ○ Haven't decided yet

14. Is research important for informing your clinical practice?

Strongly Disagree	Disagree	Neutral	Agree	Strongly Agree
○	○	○	○	○

15. Is research taught well?

Strongly Disagree	Disagree	Neutral	Agree	Strongly Agree
○	○	○	○	○

16. Overall, how enthusiastic are residents in your program about research?

Not At All	Somewhat	Enthusiastic	Very Enthusiastic
○	○	○	○

17. Overall, how would you rate the resident research productivity of your program?

Very Low	Low	Moderate	High	Very High
○	○	○	○	○

18. How would you rate your faculty's research productivity?

Very Low	Low	Moderate	High	Very High
○	○	○	○	○

19. Overall, how qualified is the faculty of your program to teach principles of research?

Not At All	Somewhat	Qualified	Very Qualified
○	○	○	○

20. Overall, how accessible is your faculty to residents seeking to do research?

Not At All	Somewhat	Accessible	Very Accessible
○	○	○	○

21. How much support does your residency program provide residents wishing to do research?

None	Some, but not enough	Supportive	Very Supportive
○	○	○	○

22. The emphasis on resident research in your department is?

None	Minimal	High	Very High
○	○	○	○

23. How would you rate your program compared to other residency programs with respect to fostering resident research?

Very Low	Low	About The Same	Higher	Much Higher
○	○	○	○	○

24. The faculty has enough time to help with research

Strongly Disagree	Disagree	Agree	Strongly Agree
○	○	○	○

25. There are sufficient faculty mentors to provide guidance for interested residents

Strongly Disagree	Disagree	Agree	Strongly Agree
○	○	○	○

26. There is sufficient technical support (ethics, biostatistics, writing, etc.)

Strongly Disagree	Disagree	Agree	Strongly Agree
○	○	○	○

27. There is a good match between resident time and researcher expectations

Strongly Disagree	Disagree	Agree	Strongly Agree
○	○	○	○

28. Where do residents in your program obtain funds to do research? (Select all that apply)

- [ ] Departmental fund for this purpose
- [ ] By cooperating with faculty who have independent grants
- [ ] By applying for grants with the aid of the program
- [ ] Must fend for themselves in finding research dollars
- [ ] Don't know

29. What types of research output does your program require? (Select all that apply)

- [ ] Original research article
- [ ] Case report
- [ ] Review article
- [ ] Present an abstract (poster or oral) at a scientific meeting
- [ ] Present an abstract (poster or oral) at rounds
- [ ] No requirement
- [ ] Don't know

30. If you have such a requirement, what actions are taken by the residency-training program if the resident research requirement is not fulfilled? (Select all that apply)

- [ ] Not applicable as we do not have a research output requirement
- [ ] Resident is not permitted to graduate
- [ ] Resident is permitted to graduate, but required to perform alternative academic work/ additional time in training
- [ ] Resident is permitted to graduate, but mention is made of failure to complete research requirement in letters of recommendation
- [ ] No policy exists regarding this contingency
- [ ] Don't know

31. If you do not have a minimum research requirement for your residents, the reasons are: (Select all that apply)

- [ ] Not applicable as we do have a minimum requirement
- [ ] Residents would object
- [ ] Faculty are not able or willing to be mentors
- [ ] Residents are too busy already
- [ ] Feel research should be optional or elective
- [ ] No way to enforce it

32. What percentage of residents in your program at each year are involved in research? (Best estimate)

Percentage (%) ____________

33. Overall, when residents develop a new research hypothesis and are active in bringing it to publication, they get adequate credit

Strongly disagree	Disagree	Agree	Strongly agree
○	○	○	○

34. Does your program have a set of rules already in place to determine such issues as (Select all that apply)

- [ ] Which name goes on the paper
- [ ] Which name goes first on a paper
- [ ] Who gets funds to present a paper at a meeting
- [ ] Don't know
